# Discrepancy of the subjective perception of the nasal appearance between independent individuals and patients undergoing functional rhinoplasty (fRPL)

**DOI:** 10.1007/s00405-022-07504-6

**Published:** 2022-06-28

**Authors:** Marleen Heiming, Eleftherios Savvas, Claudia Rudack, Markus Stenner, Christoph Spiekermann

**Affiliations:** 1 Department of Otorhinolaryngology, University Hospital Münster, Münster, Germany; 2 Medical Clinic, Deutsche Bundeswehr, Cologne, Germany

**Keywords:** Rhinoplasty, Outcome, Subjective perception, Body image, Visual analog scale

## Abstract

**Purpose:**

Satisfaction with the nasal appearance is a crucial outcome parameter in functional rhinoplasty (fRPL). The visual analogue scale is a suitable instrument not only for the preoperative patient assessment, but also as a patient-reported outcome measure in fRPL. In this study, we analyzed whether a high discrepancy in the preoperative subjective perception of the nasal appearance between patients and other individuals predicts a lower level of satisfaction with the postoperative result and hence a worse outcome of fRPL.

**Methods:**

Standardized facial pictures of patients (*n* = 80) who underwent fRPL were taken preoperatively, 3 and 12 months postoperatively. In addition, patients were asked to complete the German version of the Utrecht Outcome Assessment Questionnaire in Aesthetic Rhinoplasty (D-OAR). The standardized facial pictures of the patients were presented to surgeons as well as to examiners without a medical background, and they were asked to evaluate the patients’ nasal appearance using the visual analogue scale.

**Results:**

The external evaluation of patient’s nasal appearance was 1.7 points higher in median than the patient’s subjective perception (range −5.7–7.00). A large discrepancy between self- and external estimation significantly correlates with higher D-OAR values (*r* = 0.539, *p* < 0.001). Patients with high scores in the D-OAR trick questions, indicating a body dysmorphic disorder, show a significant larger discrepancy between the external- and the self-assessment (2.8 ± 0.5 vs. 1.4 ± 0.3, mean ± SEM, *p* = 0.017).

**Conclusions:**

Large discrepancies between the self and external assessment of the nasal appearance are associated with a high-perceived influence of the appearance of the nose on the quality of life in patients undergoing functional rhinoplasty. That might be an indicator for unrealistic expectations concerning the postoperative outcome. Knowledge about this factor helps to identify the need for intensive discussion about possibilities and limitations of the planned procedure to avoid postoperative dissatisfaction.

## Introduction

Functional rhinoplasty (fRPL) aims to improve both the functional and aesthetic aspect of the nose and has the possibility to achieve significant benefits for patients' health-related quality of life [1]. The postoperative outcome mainly depends on the subjective perception of the nasal appearance [2]. Male gender, younger age, relationship or family dysfunction, a narcissistic personality, as well as demanding and unrealistic expectations have been described to be negative predictors in fRPL [3]. It is easy to note the gender, age and various habits of a patient. Identifying and assessing more complex characteristics, such as narcissistic traits or personalities, are also possible [4]. However, a tool to determine unrealistic expectations in patients, especially those undergoing rhinoplasty, has not yet been described.

Lohuis et al. developed a short questionnaire to evaluate the influence of nasal appearance on the quality of life in patients undergoing aesthetic rhinoplasty: the Utrecht Outcome Assessment Questionnaire in Aesthetic Rhinoplasty (OAR) [5]. The questionnaire consists of a visual analogue scale (VAS) and five multiple-choice questions concerning quality of life. Preoperatively, the questionnaire reveals important aspects and possible disturbances of the body image which could be negative predictors concerning the outcome of rhinoplasty [2]. Furthermore, the questionnaire is a useful tool for patient reported outcome measure in rhinoplasty. A German version of the form has been developed and validated recently (D-OAR) [2]. High scores on the five questions indicate an important negative impact of nasal appearance on the quality of life and are associated with low VAS scores [2]. Previously, we have shown the VAS to be an adequate tool to assess the patient-reported outcome in functional rhinoplasty [6]. In the present study, we used the VAS to identify discrepancies between patients' and assessors' evaluations and wondered whether the analysis of VAS differences between self and external assessment could reveal unrealistic expectations as a negative predictor of the outcome in fRPL.

## Materials and methods

### Patients

The data of patients (*n* = 80), who underwent functional rhinoplasty between March 2015 and December 2016 in the department of otorhinolaryngology at the Münster University Hospital have been included into this clinical study. The study has been approved by the institutional ethics committee and written informed consent was obtained from all subjects.

### Questionnaire

The German version of the Utrecht Outcome Assessment Questionnaire in Aesthetic Rhinoplasty (D-OAR) consists of a VAS to determine the subjective perception of the nasal appearance with a range from 0 (very ugly) to 10 (very nice) and five questions to assess the perceived influence of the nasal appearance on the quality of life. The questions can be answered using a five-point Likert scale with a range from 1 (“not at all”) to 5 (“very much often”). A sum score of 25 points indicates a high perceived influence on the quality of life. The questions 3 and 4 are considered trick questions indicating disorders in body image perception [2]. The patients were asked to complete the D-OAR preoperatively, 3 and 12 months postoperatively. Furthermore, a five-point Likert scale was used to assess the patients’ satisfaction with the outcome in general with a range from 0 (“not satisfied”) up to 4 (“totally satisfied”).

### Photodocumentation and data acquisition

Standardized facial photographs were taken preoperatively, 3 and 12 months postoperatively from four pre-defined perspectives. These pictures were combined to a presentation. Hence, each slide comprises four standardized pictures of one patient at one time-point. Attention was paid to a re-arrangement of the pictures so that the examiners were blinded concerning the time-point of photo documentation (Table 1).

**Table 1:** Exemplary illustration of the re-arrangement of the photodocumentation within the presentation to achieve a blinding of the examiners

Slide	Patient#	Time-point of photodocumentation
Slide 1	Patient 1	3 months postoperatively
Slide 2	Patient 2	Preoperatively
Slide 3	Patient 3	12 months postoperatively
Slide 4	Patient 1	Preoperatively

Examiners without medical background (*n* = 4) as well as ENT surgeons (*n* = 2) were asked to evaluate the nasal appearance of the patients on the standardized photographs in the presentation using the same VAS as mentioned above. Results were recorded using tabular sheets.

### Statistical analysis

Results were described as mean value ± standard deviation or median with range (minimum and maximum). The association between two variables were described by report of Spearman’s rank correlation coefficient (*r*_sp_) and were considered to be excellent (*r*_sp_ > 0.8), good (0.5 < *r*_sp_ ≤ 0.8) or low (0.2 < *r*_sp_ ≤ 0.5). Student’s *t* test was performed to compare two independent groups with normally distributed data and the Mann–Whitney *U* test was used for skewed data. Linked variables were analysed by performing a paired *t* test for normally distributed or the Wilcoxon rank sum test in case of skewed data. Cut-off values were determined by Receiver Operating Characteristics (ROC) curve analysis. Discriminative power of the cut-off value was described by calculation of the *Area under the curve* (*A*) and was considered to be excellent (*A* > 0.9), good (*A* > 0.8), acceptable (*A* > 0.7) or poor (*A* > 0.6). Results with *p* > 0.05 were considered not to be statistically significant. Data were collected, analysed and artwork was created by using IBM^®^ SPSS^®^ Statistics 26 for Windows (IBM Corporation, Somers, NY, USA). Statistical advice was given by the Institute of Biometrics and Clinical Research at the University of Münster.

## Results

### Patients and examiners

Male patients (*n* = 37) with a median age of 26 years (range 15–63 years) and female patients (*n* = 43) with a median age of 26 years (range 16–70 years) were included in this single-centre study.

Male and female examiners with a median age of 35 years (range 19–58 years) (*n* = 4) and surgeons (*n* = 2) were included.

### Subjective perception of nasal appearance

**Fig. 1:**
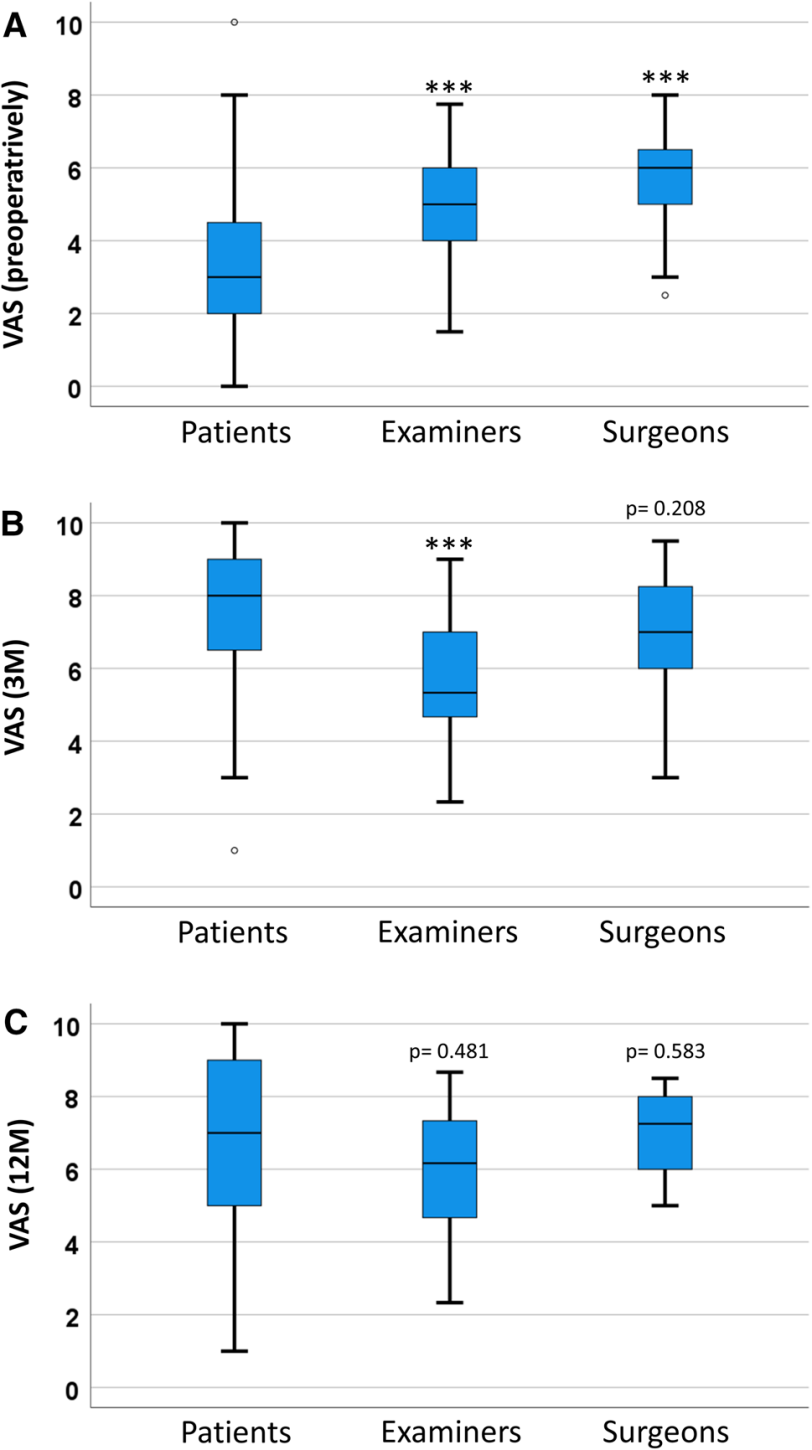
Boxplots illustrating the evaluation of the patients’ nasal appearance by patients, examiners and surgeons preoperatively (**A**), 3 months (**B**) and 12 months (**C**) postoperatively. Patients evaluated their nasal appearance significantly worse compared to examiners and surgeons. Three months after surgery patients showed significant higher VAS scores compared to examiners whereas there was no difference compared to surgeons. Twelve months after surgery, no differences between the cohorts were observable. (*VAS* visual analogue scale, *3 M* 3 months postoperatively, *12 M* 12 months postoperatively, ***p < 0.001)

Preoperatively, the nasal appearance was rated significantly worse by the patients (3.3 ± 2.0, mean ± SD) than by the examiners (5.1 ± 1.5, *p* < 0.001) and the surgeons (5.8 ± 1.1, *p* < 0.001) (Fig. 1A). A significant increase in the VAS score could be observed in all three groups 3 and 12 months postoperatively. Interestingly, the patients evaluated the nasal appearance significantly better than the examiners three months postoperatively (7.3 ± 2.2 vs. 5.7 ± 1.7, *p* = 0.001), whereas there was no significant difference between patients and surgeons (6.8 ± 1.8, *p* = 0.208) (Fig. 1B). No significant differences could be observed between the patients (6.6 ± 2.7) and the probands (6.1 ± 1.8, *p* = 0.481), or between the patients and the surgeons (7.1 ± 1.2, *p* = 0.583) 12 months after surgery (Fig. 1C). There were no differences when male examiners rated female patients or vice versa (*data not shown*).

### Discrepancy of the subjective perception of the nasal appearance

**Fig. 2:**
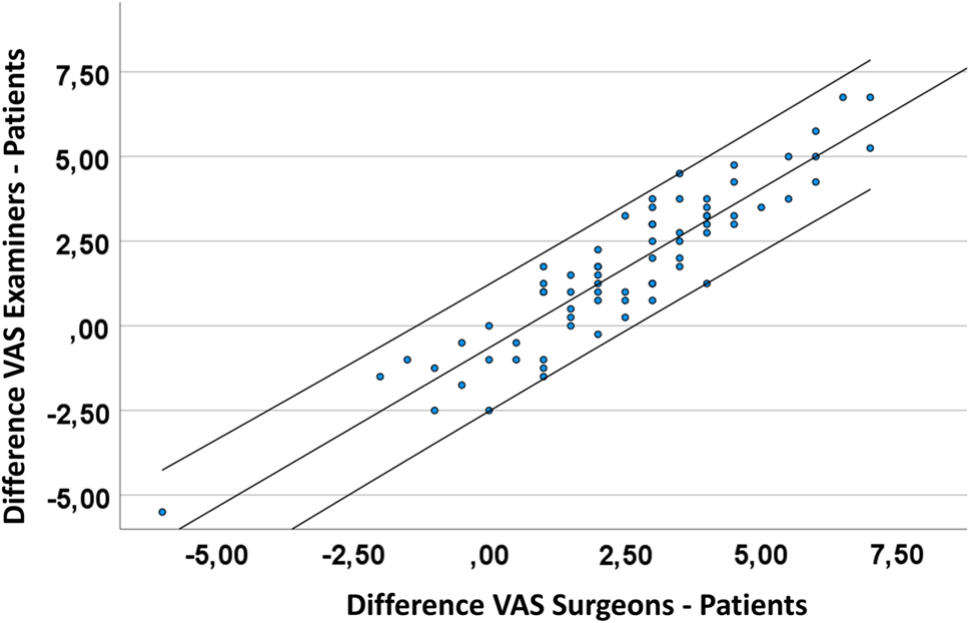
Scatter plot. The scatter plot shows graphically that the external assessment by the surgeons and the examiners is obviously higher than the self-assessment of the patients. Excellent correlations of the difference between the assessment of examiners or surgeons and patients (*r*_sp_ = 0.9, *p* < 0.001) may indicate that the assessment performed with the VAS has a good reliability

**Fig. 3:**
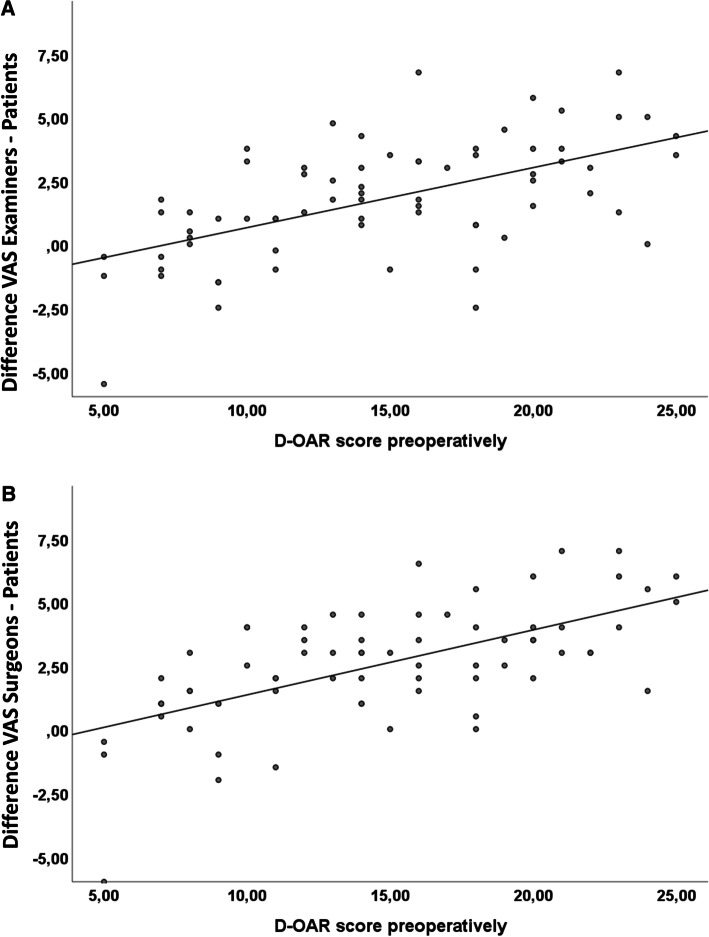
**A, B** Scatter plot With regard to the D-OAR Questionnaire, the discrepancy between self-perception and perception by others is strongly related to the perceived quality of life. The more the nose restricted the patients' quality of life (visible through higher D-OAR scores), the more significant was the difference in the VAS measured in our analysis (*r*_sp_ = 0.6, *p* < 0.001)

**Fig. 4:**
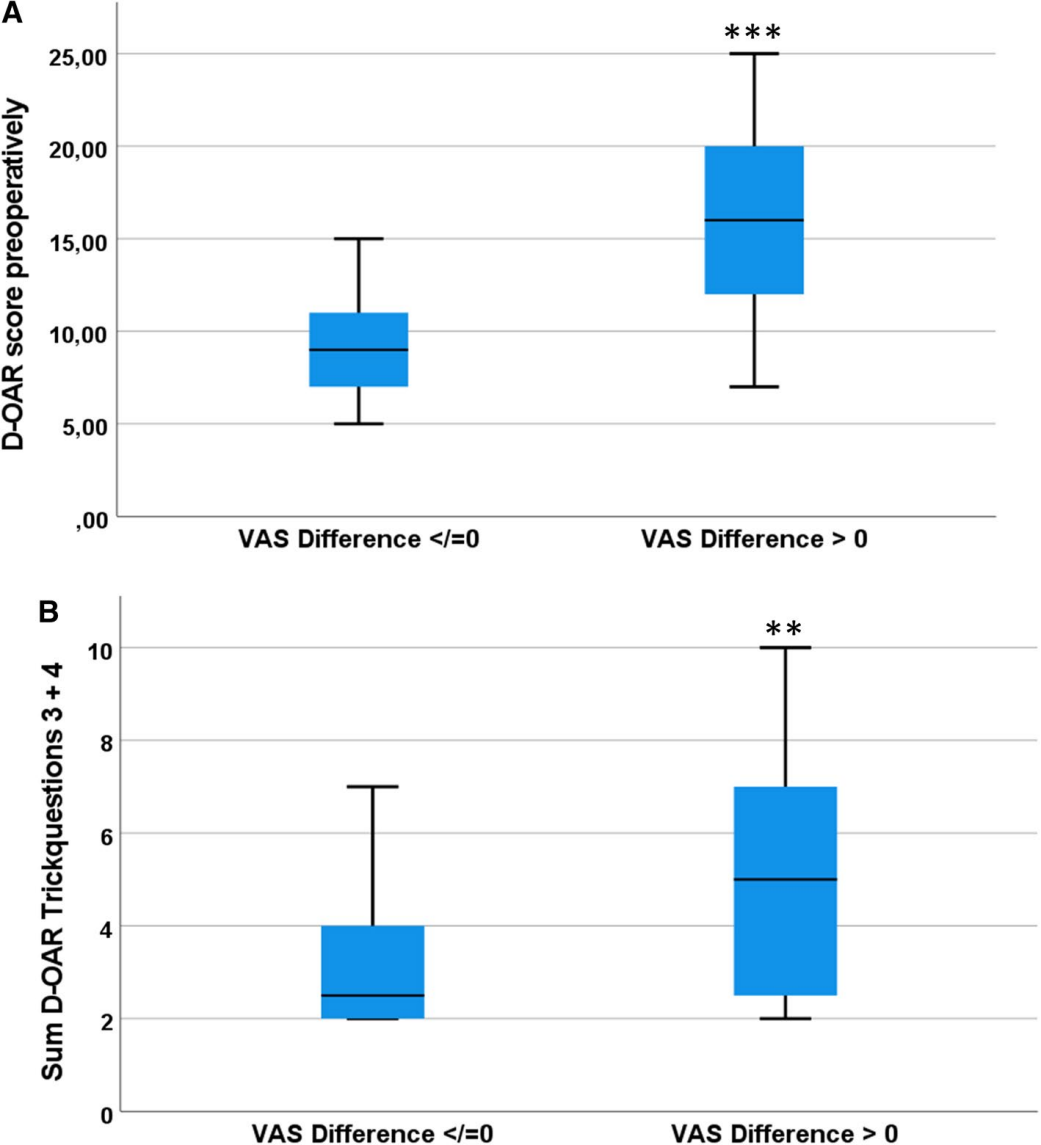
Box plot Patients with a worse assessment of nasal appearance compared to surgeons and examiners showed significantly higher preoperative D-OAR sum scores than patients with a better assessment (15.9 ± 5.1 vs. 9.7 ± 4.5, *p* < 0.001). Particularly striking was the difference between the self-perception and the perception of others with regard to the trick questions in the perceived quality of life related to the appearance of the nose. The higher the scores in the D-OAR, the higher the difference between the self- and peer-assessment of the nose (4.9 ± 2.5 vs. 3.2 ± 1.7, *p* = 0.005)

Excellent correlations of the difference between the evaluation of examiners or surgeons and patients (*r*_sp_ = 0.9, *p* < 0.001) may indicate a good reliability of the performed assessment using the VAS (Fig. 2). Larger differences of VAS values between examiners and patients are associated with higher D-OAR score values (*r*_sp_ = 0.6, *p* < 0.001). Same was observed for differences of the VAS values between surgeons and patients: the greater the difference on the VAS, the greater the perceived influence of the nasal appearance on the quality of life (*r*_sp_ = 0.6, *p* < 0.001) (Fig. 3A, B). Patients with a worse evaluation of the nasal appearance compared to surgeons and examiners showed significantly increased preoperative D-OAR sum scores to patients with a better evaluation (15.9 ± 5.1 vs. 9.7 ± 4.5, *p* < 0.001) (Fig. 4A). Furthermore, they showed significant higher sum scores of the trick questions 3 and 4 (4.9 ± 2.5 vs. 3.2 ± 1.7, *p* = 0.005) (Fig. 4B). A cut-off value of 2.75 VAS points (sensitivity 0.7, specificity 0.6, *A* > 0.7) of difference between the self- and external-estimation of the nasal appearance was determined to identify patients, who perceived a higher influence of their nasal appearance on their quality of life.

No association was found between the preoperative VAS difference and the satisfaction with the postoperative outcome (3.3 ± 0.8 vs 3.2 ± 0.4, p = 0.880) (*data not shown*).

## Discussion

In this retrospective study, we identified significant discrepancies between subjective and external perceptions of nasal appearance in patients who underwent functional rhinoplasty. Patients with lower VAS score values showed increased D-OAR score values preoperatively, indicating a higher perceived impact of nasal appearance on the quality of life.

Notably, Herruer et al. observed that patients who underwent rhinoplasty had more self-confidence issues related to their appearance than the general, unconcerned population. Patients who were less self-confident were more likely to seek rhinoplasty. Surgery then significantly improved quality of life in these patients [7].

In contrast, some patients do not benefit from rhinoplasty. In facial cosmetic surgery, *unrealistic expectations* have been found to be a negative predictor of patient satisfaction [3]. Interestingly, *Body Dismorphic Disorder* (BDD) was shown to have a negative impact on the perception not only in aesthetic but also in functional issues of the nose [8]. BDD leads to significant impairment of the patient's personal lifestyle, which may result in depressive disorder, anxiety or obsessive–compulsive behaviour. [9]. Inverse correlation was observed between BDD symptoms and postoperative satisfaction in aesthetic rhinoplasty: patients with more severe BBD symptoms were significantly less satisfied with the final postoperative outcome [10].

Although we pay attention to symptoms of BDD and refuse surgery in such cases, some patients have extraordinary expectations for the postoperative appearance of the nose, which are unrealistic and not achievable by surgery. Hence, it is important for the surgeon to uncover these concepts preoperatively and talk to the patient about possibilities and limitations to avoid upcoming postoperative dissatisfaction. Expectations such as “the operation will fix my troubles” or “the operation will make me more attractive” can lead to frustration and serious psychological decompensation and must be addressed as early as possible by an interdisciplinary team [11].

The use of the VAS allows the surgeon to record the individual perception of the patient’s nasal appearance at a glance during the outpatient management. Large differences between the patient’s and the surgeon’s VAS score may reveal a wrongly exaggerated negative perception of the nasal appearance. That could be associated with unrealistic expectations concerning the outcome or even a hint for BDD. Hence, we recommend the use of the VAS preoperatively in clinical routine practice. Discrepancies > 2 points on VAS indicate exaggerated negative perception of the nasal appearance and should be addressed prior to nasal surgery.

The VAS is a well-established assessment tool to assess the aesthetic patient-reported outcome in rhinoplasty [6]. However, the simplicity of scaling and classifying results collected with a visual analogue scale has its limitations. In a study on perceived pain intensities, Elfving et al. criticized the VAS as not suitable in assessing complex issues [12]. Hence, one could insist that the external appearance of the nose is too complex to be assessed just on the basis of the VAS. On the other hand, the VAS was described to be an instrument with an excellent test–retest reliability that is easy and rapid to complete, and only slightly susceptible to confounding bias [13, 14].

Our data revealed partial discrepancies in the absolute values of the rating by the individual persons, which, however, ultimately indicate the same tendency. This might be due to the *ideal* facial aesthetic that largely depends on the individual cultural and ethnic background [15]. In addition, differences between the patient's and the surgeon's assessment of nasal appearance may occur due to differing priorities: For the patient, nasal width is more important, whereas the surgeon’s view is focused on nasal symmetry [16].

Parsa et al. observed an influence of the age and the gender of the observer on the evaluation of the perceived benefits after aesthetic rhinoplasty with regard to the factors: attractiveness, feminity and confidence [17]. No gender-specific differences in the evaluation of the nasal appearance using the VAS were found in this study. However, further studies with more examiners of different ages and gender are necessary to confirm this data.

Although we observed high preoperative VAS differences to be associated with high D-OAR scores, there was no influence on postoperative satisfaction levels in general detectable. This could be due to the improved function, which is considered to be the most important aspect in fRPL. Furthermore, the improvement of nasal function by fRPL seems to be independent of the preoperative mental health status [18].

Among patients undergoing aesthetic rhinoplasty, however, higher preoperative aesthetic expectations are observed [19]. Therefore, further studies have to determine the influence of the VAS difference on the outcome in cosmetic rhinoplasty. Furthermore, a prospective randomized intervention study would be of great interest with an additional intensified discussion about possibilities and limitations of fRPL in dependence on VAS difference values.

## Conclusion

Large differences between the external and the self-assessment of the patient’s nasal appearance are associated with a high-perceived influence of the nasal form on the quality of life in patients undergoing fRPL and may reveal unrealistic expectations concerning the outcome. The VAS is a short and helpful tool to determine these differences during preoperative outpatient examination.
